# Interictal spike networks predict surgical outcome in patients with drug‐resistant focal epilepsy

**DOI:** 10.1002/acn3.51337

**Published:** 2021-05-05

**Authors:** Abdullah Azeem, Nicolas von Ellenrieder, Jeffery Hall, Francois Dubeau, Birgit Frauscher, Jean Gotman

**Affiliations:** ^1^ Department of Neurology and Neurosurgery Montreal Neurological Institute McGill University Montréal Quebec Canada; ^2^ Department of Neurology and Neurosurgery Montreal Neurological Institute and Hospital McGill University Montréal Quebec Canada

## Abstract

**Objective:**

To determine if properties of epileptic networks could be delineated using interictal spike propagation seen on stereo‐electroencephalography (SEEG) and if these properties could predict surgical outcome in patients with drug‐resistant epilepsy.

**Methods:**

We studied the SEEG of 45 consecutive drug‐resistant epilepsy patients who underwent subsequent epilepsy surgery: 18 patients with good post‐surgical outcome (Engel I) and 27 with poor outcome (Engel II–IV). Epileptic networks were derived from interictal spike propagation; these networks described the generation and propagation of interictal epileptic activity. We compared the regions in which spikes were frequent and the regions responsible for generating spikes to the area of resection and post‐surgical outcome. We developed a measure termed source spike concordance, which integrates information about both spike rate and region of spike generation.

**Results:**

Inclusion in the resection of regions with high spike rate is associated with good post‐surgical outcome (sensitivity = 0.82, specificity = 0.73). Inclusion in the resection of the regions responsible for generating interictal epileptic activity independently of rate is also associated with good post‐surgical outcome (sensitivity = 0.88, specificity = 0.82). Finally, when integrating the spike rate and the generators, we find that the source spike concordance measure has strong predictability (sensitivity = 0.91, specificity = 0.94).

**Interpretations:**

Epileptic networks derived from interictal spikes can determine the generators of epileptic activity. Inclusion of the most active generators in the resection is strongly associated with good post‐surgical outcome. These epileptic networks may aid clinicians in determining the area of resection during pre‐surgical evaluation.

## Introduction

Surgery is a common treatment option for patients with drug‐resistant epilepsy.^1^ Surgery involves resection of the region responsible for seizure generation, the epileptogenic zone (EZ).^2^ Multiple modalities are used to localize the EZ, one of which is stereo‐electroencephalography (SEEG). Stereo‐electroencephalography records brain activity using implanted depth electrodes in an attempt to localize the region where seizures originate.^1^, ^3^ However, seizures are not always localized to a specific region, as epileptic activity propagates to distant regions.^4^, ^5^ Even in cases where epileptic activity seems to be localized, resection of the predicted EZ may not result in seizure freedom.^1^ Between seizures, patients also present brief EEG events called interictal spikes, which have been shown to propagate across the cortex.^6^, ^7^ Recording interictal spikes requires only a few hours, whereas recording seizures requires several days of hospitalization. Improved understanding of spike propagation led to the emerging view of the epileptic focus as the main node in an overarching network.^8^ Though several research groups have explored network connectivity in epilepsy, the subject remains incompletely understood.

Using SEEG, we investigated epileptic networks derived from interictal spike propagation. The two aims of this study were to (i) delineate an epileptic network derived from interictal spike propagation recorded on SEEG and (ii) explore the association between nodes of the epileptic network and the area of resection during epilepsy surgery. We hypothesized that inclusion in the resection of areas responsible for *generating* interictal spikes would be associated with good post‐surgical outcome, and this may assist surgeons in localizing the EZ.

## Methods

### Population

We identified consecutive patients from the SEEG database at the Montreal Neurological Institute (MNI), between 2010 and 2015 who met the following inclusion requirements: (i) at least 3 days of SEEG recording (to minimize any effects of anesthesia or acute effects of implantation) (ii) resective epilepsy surgery; (iii) pre‐surgical, peri‐implantation, and postoperative brain imaging; (iv) 1‐year postoperative outcome scored using Engel classification (class I, good outcome; class II–IV, poor outcome).

### SEEG recording and segment selection

Patients underwent SEEG exploration as per the routine clinical procedure, following an inconclusive non‐invasive evaluation. Intracerebral electrodes (DIXI Medical, Besancon, France; or manufactured on‐site) were stereotactically implanted using an image‐guided system (SSN Neuronavigation System) with or without robotized surgical assistant (ROSA; Medtech, Montpellier, France).^9^ Areas of implantation were determined according to clinical data that defined suspected epileptic regions. SEEG recordings were band‐pass filtered at 0.3–500 Hz and sampled at 2000 Hz; recordings were done using the Harmonie EEG system (Stellate, Montreal, QC, Canada). Review for artifacts and spike detection were done using a bipolar montage.

Two hours of continuous awake interictal activity were clipped from a recording ~ 72 h post‐implantation. Previous literature suggests that the effects of anesthesia or acute effects of electrode placement are minimized 72 h post‐implantation.^11^ It was demonstrated that patient‐specific interictal spike propagation patterns are consistent across multiple 30‐min segments including different stages of vigilance.^10^ The 2‐h recordings were split in two 1‐h epochs. Analysis was run separately for each epoch. Results from the second epoch were used exclusively to test the predictive ability of our methods.

### Spike detection

Interictal epileptic discharges (IEDs) were detected using a modified version of an algorithm from Janca et al.^12^ The algorithm was modified such that it did not down‐sample the data to 200 Hz; rather, the data were analyzed at the recorded 2000 Hz. Removing down‐sampling retained temporal resolution at 0.5 ms. A modification was made to eliminate false detections caused by rhythmic bursts: if the probability of IED detection was greater than 90% across more than four consecutive 120‐ms segments, these events were classified as burst activity, not as IEDs. The algorithm detects the peak of IEDs (accuracy is low when trying to detect IED onset).

### Spike propagation

To determine spike propagation between two channels, we tested for significant delays between a pair of channels as described below. Once we established propagation between two channels, average latency was used to determine the direction of propagation. This allowed us to construct an epileptic network that described the generation and propagation of spikes between sources.

**Figure 1:**
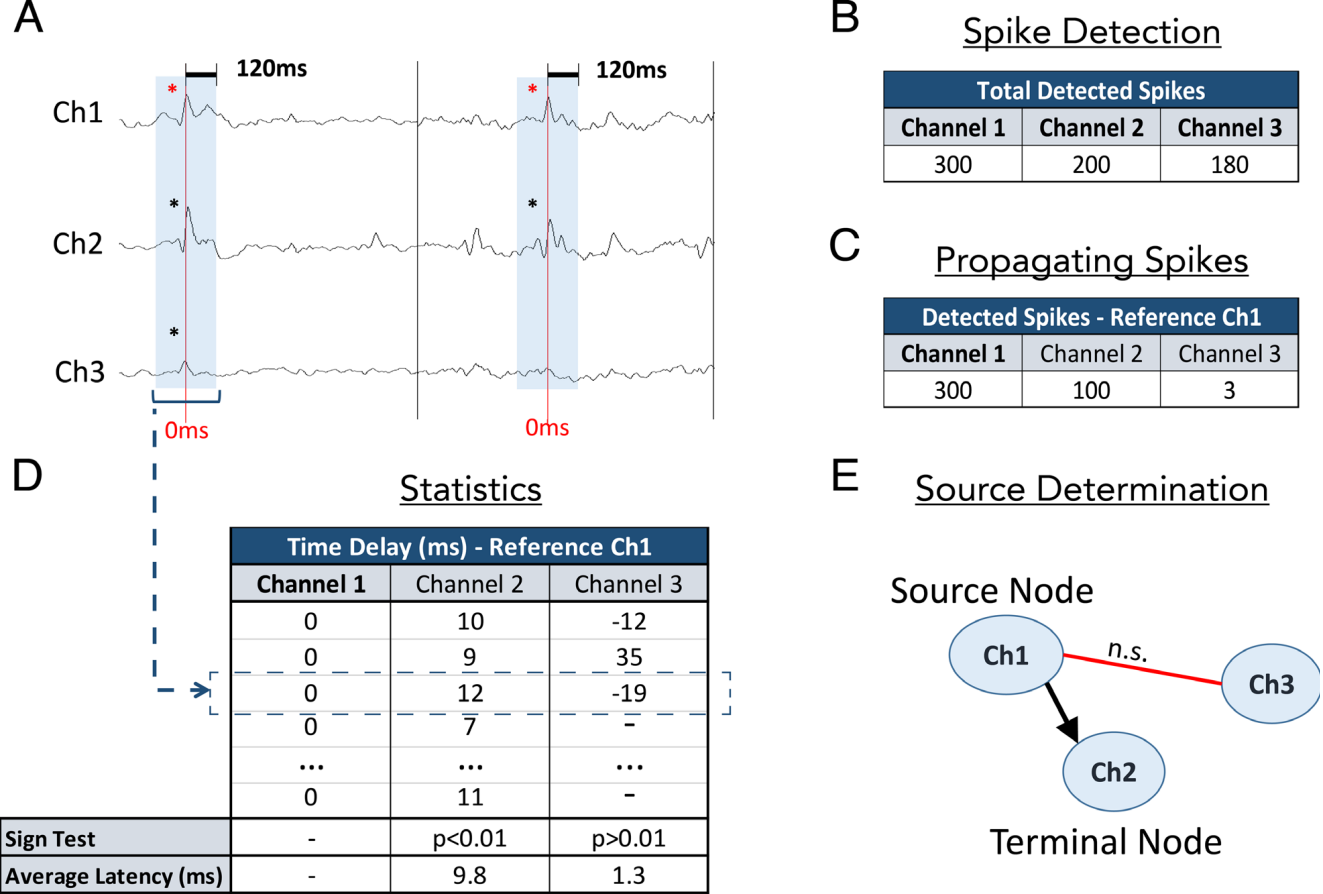
Propagation network construction. (A) A sample SEEG recording from a patient with three channels. Spikes are denoted by asterisks. (B) Using a spike detection algorithm, we detect the total number of spikes at all channels. (C) Taking turns, we treat each channel as a reference. In this example we only show Ch1 as a reference. The spikes in the reference channel are called initial spikes (denoted by red asterisks in Fig. 1A). We then count the number of spikes in other channels that fall within 120 ms before or after each initial spike; these spikes are called propagating spikes (denoted by black asterisks in Fig. 1A). (D) For each channel, we list the latency (ms) between the propagating spikes and initial spikes. The sign test is used to determine whether spikes on a given channel occur with consistent positive or negative time delay with respect to spikes on the reference channel (null hypothesis). The positive sign test between Ch1 and Ch2 suggests that there is directional propagation between these channels. The average latency between Ch1 and Ch2 (9.8 ms) suggests that spikes in Ch2 tend to occur after spikes in Ch1. There is no propagation relationship between Ch1 and Ch3. (E) Propagation map showing the significant propagation from Ch1 to Ch2, and the lack of propagation between Ch1 and Ch3. In this example Ch1 is a source node (an area from which spikes propagate to other regions but does not receive propagation), and Ch2 is a terminal node (an area that receives propagation from other regions but does not propagate spikes further). The relationship between Ch2 and Ch3 is not explored in this example

Previous studies suggest maximum spike propagation times of ~ 100 ms from temporal to frontal regions, and we used a 120‐ms window to ensure enough time for propagation.^13^ Within a channel, spikes following another spike by less than 120 ms were excluded from analysis. The process of determining propagation is described in Fig. 1. Each channel was treated as a *reference channel*, where spikes occurring in that channel were named “initial spikes” at *t* = 0 ms; spikes from all other channels within 120 ms (before and after) of each initial spike were considered to be “propagating spikes,” and their latency from the initial spike was recorded. If the latency was 3 ms or less, the two spikes were said to occur simultaneously, and the latency was set to 0 ms. The one‐sample sign test (α=0.01) was used to determine whether spikes on a given channel occur without a consistent positive or negative delay with respect to the reference channel (null hypothesis). Rejection of the null hypothesis suggests a statistically significant and directional time relationship between two channels. We consider a significant time relationship between any two channels as indicative of temporal propagation. The direction of propagation was determined by the mean latency between the spikes in the two channels; we thus determined in which of the two channels spikes occur first on average. The process is repeated, taking in turn every channel as a reference channel, such that all channels have eventually been compared to each other.

### Constructing the epileptic network

Once we determined the pairs of channels that show consistent directional spike propagation, we constructed a network in which each node is a channel classified according to propagation patterns (Fig. 1). There are three categories of nodes: *source nodes*, which are nodes from which spikes propagate but which do not receive propagation from other nodes; *intermediate nodes*, which both receive and generate propagation; and *terminal nodes*, which only receive propagation. These nodes were used to construct propagation maps. All spikes detected at source nodes are referred to as *source spikes*. Networks are constructed twice for each patient; once using the first 1‐h SEEG epoch and again using the second 1‐h SEEG epoch.

### Comparison of epileptic network properties with area of resection and surgical outcome

**Figure 2:**
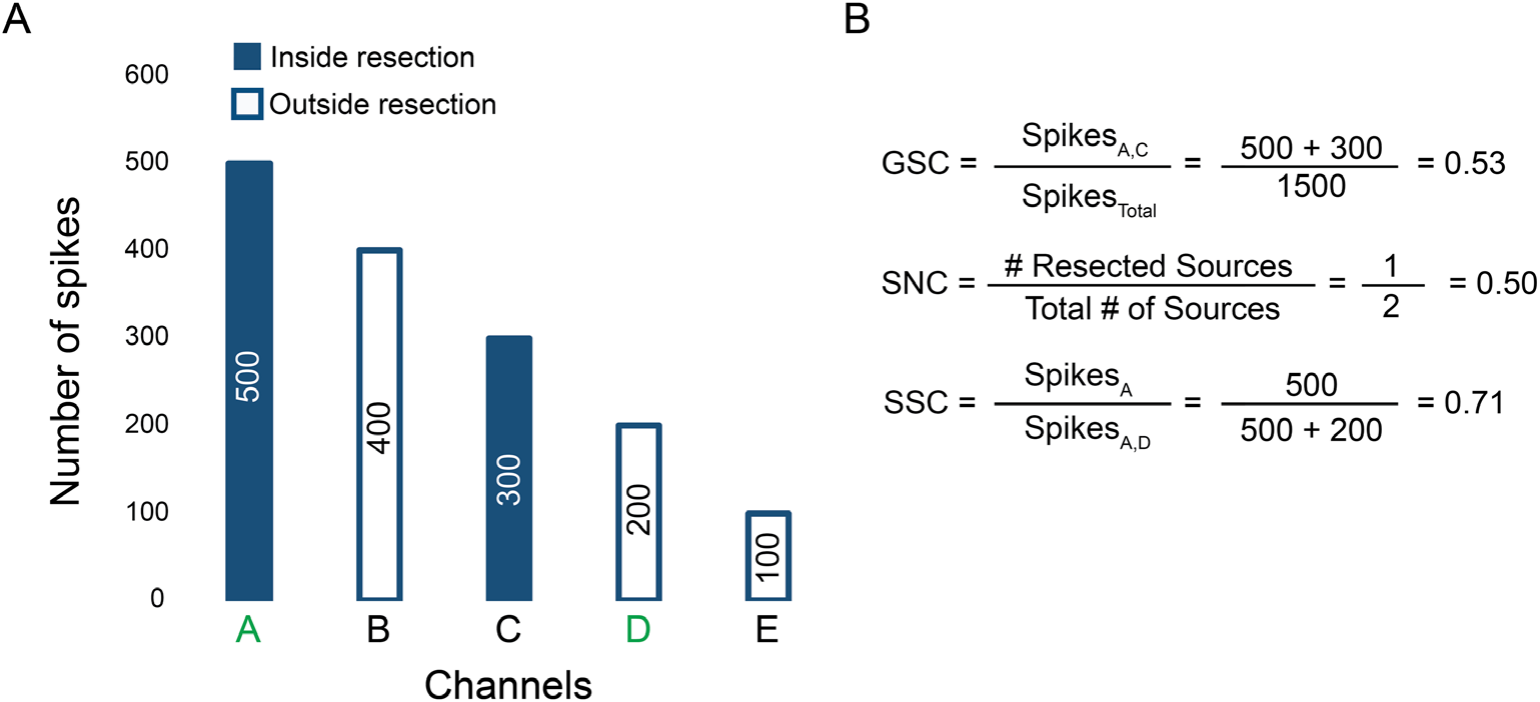
Calculation of concordance measures. (A) Total number of spikes detected for an example patient with five SEEG channels. Source node channels are green. (B) The calculation of our three measures: general spike concordance (GSC), which measures the proportion of spikes in the resection (without considering propagation); source node concordance (SNC), which measures the proportion of source nodes in the resection; and source spike concordance (SSC), which measures the proportion of source spikes (spikes detected at source nodes) in the resection

Resections were performed independently of this analysis. Since there exists no direct method to observe the EZ, we use information on post‐surgical outcome to deduce whether the EZ was included in the resection. For patients with good outcome, we assume that seizure freedom suggests that the EZ was included in the resection. For patients with poor outcome we assume that the EZ was not included in the resection, since these patients continue to have seizures post‐surgery. To determine the impact of having resected certain nodes in an epileptic network (defined by spike propagation) and whether inclusion in the resection of certain nodes could predict surgical outcome, we defined three measures: general spike concordance, source node concordance, and source spike concordance (Fig. 2).

*General spike concordance* measures whether inclusion of the most epileptically active channels in the resection is associated with outcome; it was calculated by dividing the number of spikes detected in resected areas by the total number of detected spikes for each patient. This measure does not take into consideration spike propagation and therefore ignores the network.

*Source node concordance* measures whether inclusion of source nodes in the resection is associated with outcome; it was calculated by dividing the number of source nodes in resected regions by the total number of source nodes for each patient.

Lastly, *source spike concordance* integrates propagation information with amount of epileptic activity. *Source spike concordance* measures whether inclusion of the most epileptically active source nodes in the resection is associated with outcome; it was calculated by dividing the number of source spikes in resected regions by the total number of source spikes, for each patient. We determined the ability of each measure to predict surgical outcome. We also considered the practicality of each measure for pre‐surgical evaluation and prediction of the EZ.

### Statistics

The one‐sample sign test (α=0.01) is a non‐parametric test that was used to determine whether spikes on a channel occur simultaneously with spikes on the reference channel (null hypothesis). The sign test has been used to assess the presence of a time delay between IEDs.^14^ The data were corrected for multiple comparisons using Bonferroni correction; for a given reference channel the number of comparisons was equal to the number of channels that had interictal spikes occurring within 120 ms of spikes on that channel. The sign test was chosen because it does not assume normal distribution. It requires consistent direction of delay in a sufficiently large number of samples to prove significance. The Anderson–Darling test was used to determine whether categorical data sets were normally distributed; these categorical data refer to comparisons of age at recording, general spike concordance, source node concordance, source spike concordance, number of significant propagation pairs, source nodes, intermediate nodes, and terminal nodes. The Wilcoxon rank sum test (α=0.05; two‐tailed) was used for comparison of non‐normally distributed categorical data. Chi‐square test (α=0.05) was used to determine whether location of the resection was associated with surgical outcome.

To minimize overfitting, the three measures of concordance defined above were cross‐validated using the first 1‐h epoch from all patients. Specifically, we used two‐fold cross‐validation for a total of 10,000 iterations. For each iteration, patients were randomly assigned to either the training set or validation set. The size of each set was consistent for each iteration, with half (48.7%) of the patients in the training set and half in the validation set. For each iteration, Youden's J statistic (informedness) was used to define the optimal thresholds (alpha values) for all concordance measures. Informedness estimates the probability of an informed decision, treating false positives and false negatives equally; informedness was calculated as sensitivity+specificity‐1. For each iteration, the optimal alpha value for the training set was applied to the validation set to calculate mean values and the distributions for performance metrics (sensitivity, specificity, positive and negative predictive values, and accuracy). Even though cross‐validation provides a robust evaluation, we also assessed performance using the alpha thresholds determined by cross‐validation, on the test set (networks built using the second 1‐h epoch).

McNemar's test was used to determine whether a concordance measure was superior to the rest for predicting surgical outcome.

## Results

### Population

From 138 patients who had undergone SEEG between 2010 and 2015, 45 fit our inclusion criteria. Of these, 6 did not have enough IEDs (over 1 h of wakefulness) from which we could detect significant propagation using our methodology; these patients were excluded. Of the remaining 39 patients, 17 were in the *good outcome* group (41% female; Engel Class I), and 22 belonged to the *poor outcome* group (59% female; Engel Class II–IV). Mean age at recording was 31 ± 11 years in the good outcome group and 33 ± 8 years in the poor outcome group (*p* = 0.608). Patient demographics and pathology can be found in Table 1. Resection location (at lobar level) was not associated with outcome (*p* = 0.283).

**Table 1:** Patient information

Patient Number	Age at SEEG Recording (Years)	Sex	Engel Outcome	SEEG Length (Minutes)	Age at Epilepsy Onset (Years)	Pathology
**Good Outcome**						
1	26	F	I	30	14	FCD (non‐specified)
2	16	M	I	30	4	FCD 2A
3	22	M	I	30	9	FCD 2B
4	28	F	I	30	8	FCD 2B
5	42	M	I	30	6	FCD 2B
6	22	M	IA	30	5	HS
7	39	M	I	30	17	FCD 2B
8	35	M	I	30	10	FCD 2A
9	36	M	I	30	16	FCD 2A
10	37	M	I	30	5	FCD 2B
11	55	F	I	30	5	HS
12	43	F	I	60	28	FCD 2A
13	21	F	I	30	12	FCD 2B
14	36	M	I	60	20	PNH
15	26	F	I	30	12	FCD (non‐specified)
16	32	F	I	60	17	FCD 3D
17	14	M	I	30	3	FCD 2B
Poor Outcome						
18	26	F	III	60	1	Non‐specific
19	43	F	IVA	30	30	Gliosis
20	39	F	IIB	30	8	FCD 2A
21	35	M	IVA	30	7	FCD 2A
22	53	F	IVA	60	14	Ganglioma
23	26	F	IIIB	30	7	Gliosis
24	29	F	III	30	15	FCD 2A, HS
25	47	M	IVB	30	0.5	FCD2A
26	29	F	IVA	30	21	FCD 2A
27	20	M	IIIB	30	9	Gliosis
28	22	F	IVA	30	12	PNH
29	22	F	IIIA	30	17	HS
30	37	M	IVB	30	18	Gliosis
31	33	F	IIIA	30	18	Gliosis
32	38	M	IIA	30	8	FCD 1B
33	37	F	II	30	27	FCD (non‐specified)
34	35	M	IIB	30	19	FCD 2A
35	23	F	IVB	30	18	FCD 2B
36	27	F	IVB	30	9	Gliosis
37	33	M	IVB	30	10	FCD 2B
38	35	M	IIIA	30	30	FCD 2A
39	30	M	IVB	30	8	Gliosis

Abbreviations: FCD, focal cortical dysplasia; HS, hippocampal sclerosis; PNH, periventricular nodular heterotopia.

### Network characteristics

**Figure 3:**
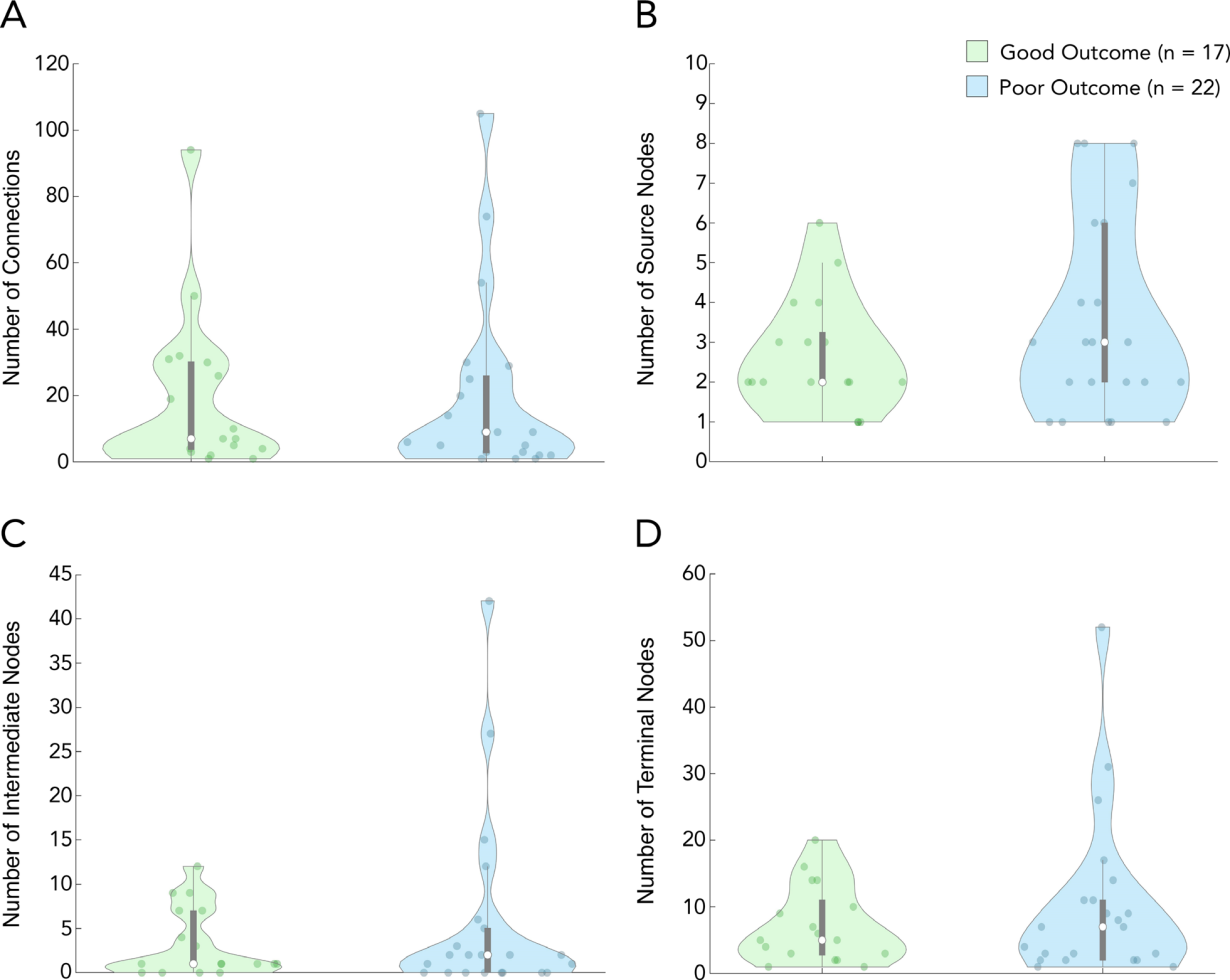
Propagation network characteristics. Characteristic features of epileptic networks. (A) Mean number of significant channel pairs, denoting a pathway of propagation; there was no significant difference between the good and poor outcome groups (*p* = 0.955). (B) Mean number of source nodes; patients in the good outcome group had significantly fewer source nodes than patients in the poor outcome group (*p* = 0.423). (C) Mean number of intermediate nodes; there was no significant difference between patients in the good and poor outcome groups (*p* = 0.897). (D) Mean number of terminal nodes; there was no significant difference between patients in the good and poor outcome groups (*p* = 0.776). Data shown is from the first 1‐h epoch for all patients. Each white dot represents group median, and gray bars represent interquartile range

Network characteristics are reported for IED networks derived from the first 1‐h epoch. Overall, patients had IEDs detected on an average of 64 ± 20 electrode contacts. As for network structure, there was no difference in the number of connections (channel pairs with a significant sign test) between the good (mean = 19 ± 24 pairs, *n* = 17) and the poor outcome group (mean = 29 ± 59 pairs, *n* = 22; *p* = 0.955). Focusing on network makeup, there was no significant difference in the number of source nodes between the good (mean = 2.5 ± 1.4 nodes, *n* = 17) and the poor outcome groups (mean = 3.0 ± 2.5 nodes, *n* = 22; *p* = 0.423; Fig. 3). There was no difference in the number of intermediate nodes between the good (mean = 3.4 ± 3.9 nodes, *n* = 17) and the poor outcome groups (mean = 5.3 ± 10.4 nodes, *n* = 22; *p* = 0.897; Fig. 3). Lastly, there was no difference in the number of terminal nodes between the good (mean = 7.2 ± 5.8 nodes, *n* = 17) and the poor outcome groups (mean = 9.9 ± 12.3 nodes, *n* = 22; *p* = 0.776; Fig. 3).

### General spike concordance

**Figure 4:**
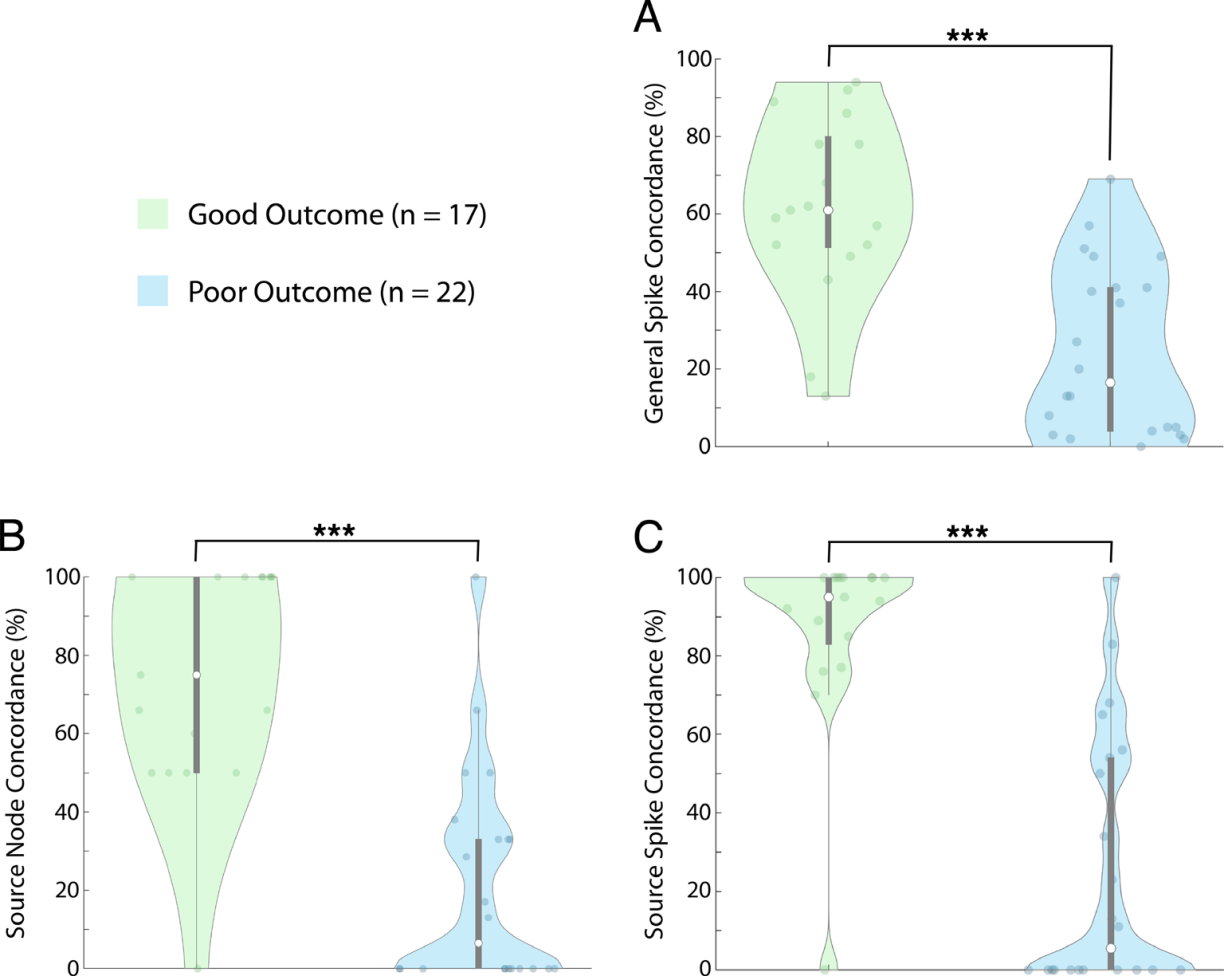
Comparing concordance measures between good and poor outcome patients. Comparison of the three concordance measures between patients in the good outcome group and patients in the poor outcome group. (A) General spike concordance compared between the good outcome groups and poor outcome group (*p* < 0.001). (B) Source node concordance compared between the good outcome group and poor outcome group (*p* < 0.001). (C) Source spike concordance compared between the good outcome group and poor outcome group (*p* < 0.001). Data shown are from the first 1‐h epoch for all patients. Each white dot represents group median, and gray bars represent interquartile range

Patients in the good outcome group showed significantly higher general spike concordance with the resection (mean = 62.7 ± 24.5%, *n* = 17) than those with poor outcome (mean = 24.5 ± 22.0%, *n* = 22; *p* < 0.001; Fig. 4). As a result of cross‐validation, we determined general spike concordance = 46% as the threshold that maximizes informedness (Youden's J statistic), that is, the separation between good and poor outcome groups. Given a threshold of 46%, general spike concordance achieved a sensitivity of 82% and specificity of 73% when evaluated using the test set. On average, 9.6 ± 4.2 nodes with the most spikes would need to be included in the resection in order to reach the 46% general spike threshold. Means and standard deviations of performance metrics from the validation set are reported in Table 2. Performance metrics are similar in the validation and test sets.

**Table 2:** Cross‐validation section and results for the test set

Cross‐validation	General Spike Concordance	Source Node Concordance	Source Spike Concordance
Sensitivity	0.80 ± 0.13	0.83 ± 0.14	0.90 ± 0.10
Specificity	0.82 ± 0.11	0.90 ± 0.07	0.93 ± 0.08
Positive Predictive Value	0.79 ± 0.09	0.89 ± 0.08	0.92 ± 0.08
Negative Predictive Value	0.84 ± 0.09	0.88 ± 0.10	0.92 ± 0.08
Accuracy	0.75 ± 0.07	0.80 ± 0.06	0.84 ± 0.05
Alpha	0.46 ± 0.08	0.48 ± 0.05	0.70 ± 0.07

Test Set	General Spike Concordance	Source Node Concordance	Source Spike Concordance
Sensitivity	0.82	0.88	0.91
Specificity	0.73	0.82	0.94
Positive Predictive Value	0.70	0.79	0.89
Negative Predictive Value	0.84	0.90	0.95
Accuracy	0.77	0.85	0.92

Note

Cross‐validation section (top): Results of 2‐fold cross‐validation for the general spike concordance, source node concordance, and source spike concordance measures; all values are presented as mean ± standard deviation. Test set section (bottom): Results for the test set.

### Source node concordance

With respect to the percent of source nodes resected (irrespective of spike rate), patients with good outcome had 3.5 times higher number of resected source nodes (mean = 74.5 ± 29.1%, *n* = 17) than those with poor outcome (mean = 20.9 ± 27.3%, *n* = 22; *p* < 0.001; Fig. 4). As a result of cross‐validation, we determined source node concordance = 48% as the threshold that maximizes informedness; sensitivity was 88% and specificity 82% when evaluated using the test set. Means and standard deviations of performance metrics calculated using the validation set are reported in Table 2. Performance metrics are similar in the validation and test sets.

### Source spike concordance

**Figure 5:**
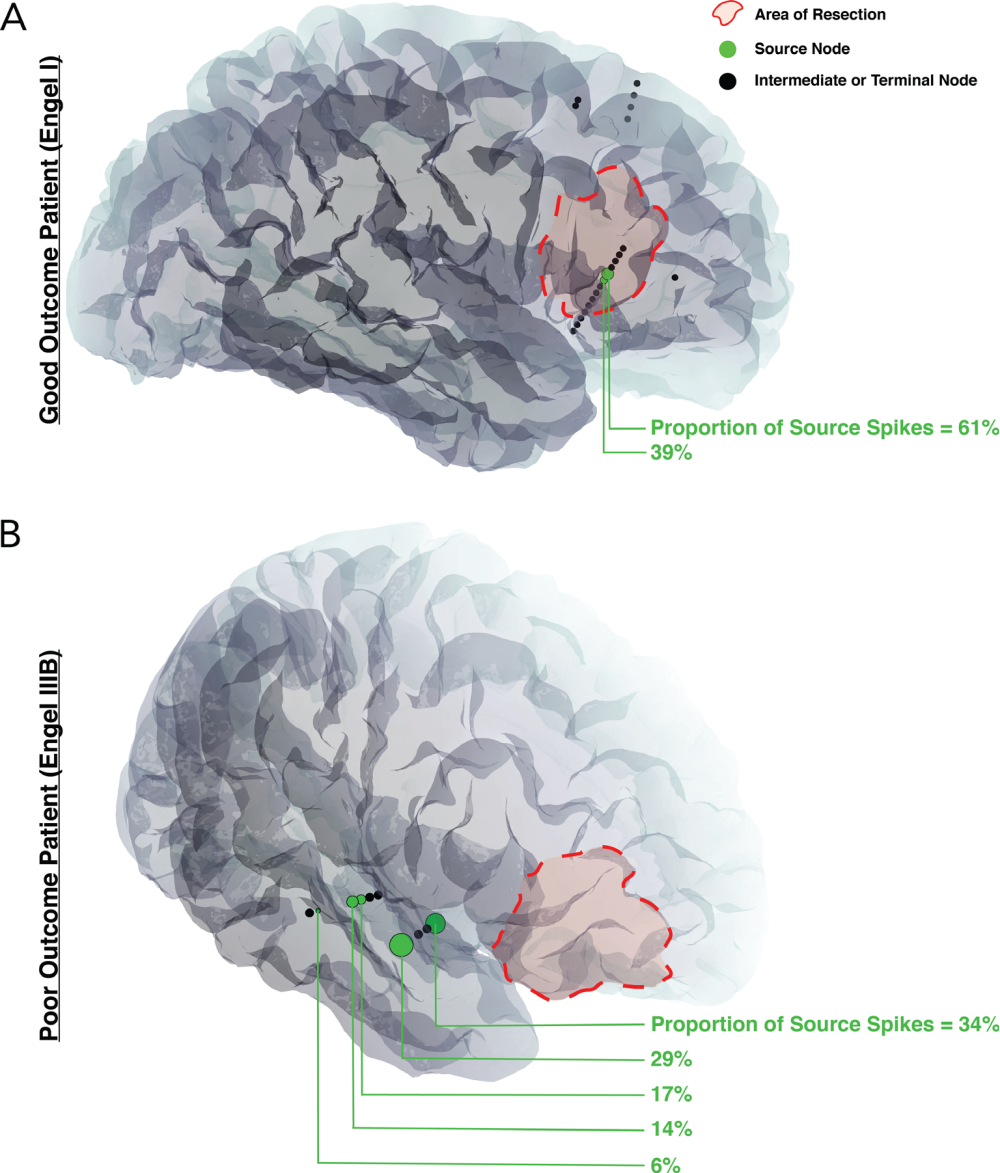
Patient‐specific imaging with an overlay of network nodes. Only the SEEG electrode contacts involved in the patient's spike propagation network are shown; spiking regions that failed to demonstrate statistically significant propagation patterns are not displayed. (A) Seizure‐free patient (Engel I) with two source nodes, both included in the resection, for a total source spike concordance value of 100%. (B) Seizure‐persistent patient (Engel IIIB) with five source nodes, none being included in the resection, for a total source spike concordance value of 0%. Interictal spikes were present in the frontal lobe, but they did not demonstrate statistically significant propagation patterns

Patients with good surgical outcome also had higher source spike concordance (mean = 87.0 ± 24.5%, *n* = 17) compared to those with poor outcome (mean = 25.3 ± 32.6%, *n* = 22; *p* < 0.001; Fig. 4). After cross‐validation, source spike concordance proved to be our most reliable measure of prediction. As a result of cross‐validation, we determined source spike concordance = 70% as the threshold that maximizes informedness; sensitivity was 91%, and specificity was 94% with the test set, and other statistics are given in Table 2. This indicates that if channels representing at least 70% of the spikes in source channels are part of the resection, there is a very high probability that the patient will have a good outcome, and conversely, if less than 70% of the spikes in source channels were resected, a poor outcome was likely. On average, the 1.5 ± 0.8 source nodes with the most spikes would need to be included in the resection to reach the 70% source spike threshold for a given patient. Using source spike concordance, there are significantly fewer nodes (1.5 ± 0.8 channels) that must be included in the resection compared to using general spike concordance (9.6 ± 4.2 channels; *p* < 0.0001) in order to meet the optimal threshold. We illustrate the concept of source spike concordance with two patients in Fig. 5. Mean values and standard deviations of performance metrics from the validation set are reported in Table 2. Performance metrics for source spike concordance are similar between the validation and test sets.

### Superiority of concordance measures

As per the McNemar test, there was no single concordance measure that was statistically superior to the others. While the predictability measures of sensitivity and specificity trend higher for source spike concordance, there was no statistically significant difference in the accuracy between source spike concordance and source node concordance (*p* = 0.180) or general spike concordance (*p* = 0.500). There was also no difference in accuracy between source node concordance and general spike concordance (*p* = 0.508). However, we consider the practicality of these three measures as it pertains to their use prospectively in clinical settings in the discussion below.

## Discussion

Large meta studies suggest the success rates of epilepsy surgery to be moderate, with 52–66% of patients achieving seizure freedom.^3^, ^15^, ^16^ Our primary aim was to design a method to increase the predictability of seizure freedom post‐epilepsy surgery; our secondary aim was to provide a method that better localizes the EZ. Our work leverages the temporal resolution of SEEG to differentiate spiking regions that initiate interictal epileptic activity from spiking regions where interictal activity results from propagation. We find that including in the resection regions that initiate interictal activity is associated with good outcome (Engel I), and we refer to these regions as source nodes in our epileptic networks. The extent of the resection of the specific source nodes with high spike rate may predict surgical outcome in patients with drug‐resistant focal epilepsy.

Epilepsy is increasingly studied as a network disorder.^17^, ^18^ A common approach to the construction of epileptic networks is through functional connectivity, which uses signals from many sources (fMRI, MRI, EEG, SEEG etc.). Networks defined by fMRI use blood oxygen level dependent (BOLD) signals, which are not directly related to the electrophysiological properties of epileptic activity and are affected by non‐epileptic activity.^19^ In contrast, using SEEG, we directly assess epileptic activity (IED occurrence) and subsequently delineate epileptic networks based on the propagation of IEDs. Indeed, the ability of IEDs to identify key epileptic regions (EZ and seizure onset zone) has been demonstrated.^7^, ^20^ This idea was first introduced by Jasper et al., who suggested that IEDs can be differentiated into “primary spikes” and spikes that are a result of propagation, with the former having more value in localizing the pathological region.^21^ The use of interictal activity for constructing epileptic networks has already been established; with the added potential to save time for patients in monitoring units, IED‐based epileptic networks may be valuable additions to surgical planning.^7^, ^22^, ^23^

The epileptic networks in this study are derived from spike propagation patterns. We interpret IEDs occurring with a consistent time difference between two contacts as indicative of a propagation relationship between IEDs at the two contacts. The sign of a consistent time difference determines the direction of propagation. Source nodes are responsible for generating interictal spikes, which then propagate to intermediate or terminal nodes. Intermediate nodes receive propagation and are involved in propagating interictal spikes to other nodes, and terminal nodes are the end‐receivers of interictal spikes. There were no differences between groups in the average size of the network (number of involved nodes), the number of source, intermediate, or terminal nodes.

We find that patients in the good outcome group showed higher general spike concordance than those in the poor outcome group, and we determined that inclusion in the resection of channels representing at least 46% of interictal spikes was correlated with good post‐surgical outcome (sensitivity 0.82 and specificity 0.73; Table 2). These findings suggest that failing to resect channels that represent > 46% of the total number of spikes will likely result in poor outcome. If planning surgery using general spike concordance, channels representing at least 46% of all spikes would need to be included in the resection for the best chance at seizure freedom. One approach is to start with the resection of the most active channel and, in a descending order of channel activity, continue to resect channels until resected channels represent at least 46% of all spikes.

While source node concordance (sensitivity = 0.88, specificity = 0.82; Table 2) has higher trending, predictability metrics than general spike concordance; it is difficult to use prospectively. For example, one of the good outcome patients had seven source nodes; to reach the 48% source node concordance threshold, four nodes would need to be included in the resection; however, based on source node concordance alone, we cannot say which four of the seven nodes to choose.

Source spike concordance may be a better clinical tool than general spike concordance and source node concordance. We achieved high levels of sensitivity and specificity when using source spike concordance (sensitivity = 0.91, specificity = 0.94; Table 2). The higher trending predictability metrics of source spike concordance may demonstrate the value of combining information about a node's spike rate with information about the node's role in the network. We find that inclusion in the resection of source nodes that contribute to at least 70% of source spikes is strongly associated with seizure freedom. More importantly, the source spike concordance measure provides a clinically practical approach for the pre‐surgical determination of the epileptic zone: include in the resection the source nodes with the highest spike rates until at least 70% of source spikes are included. Despite the similarities between the general spike concordance and the source spike concordance approaches, the source spike concordance requires much fewer channels in the resection (1.5 vs. 9.6). This may be due to the fact that when using source spike concordance, the resection may only need to include regions that generate source spikes, whereas, when using general spike concordance, many high‐activity channels may be the result of propagating spikes, but these are not separated from source spikes with this measure. As a result, source spike concordance may ensure the removal of true sources of epileptic activity while leaving downstream regions in the epileptic network intact.

There are benefits to using IED‐based networks over seizure networks. First, waiting to record a spontaneous seizure can be time consuming; one study found the average length of stay to be 7 days in their invasive monitoring unit.^24^ Recording of interictal activity, on the other hand, only requires a few hours of EEG monitoring. Second, the identification of the seizure onset zone is difficult, and while quantitative methods exist, some only apply to certain seizure types and their ability to predict surgical outcome has not been demonstrated.^25^, ^26^, ^27^ In contrast, we propose a simple quantitative method to delineate the source of interictal activity, and we demonstrate a strong ability to predict surgical outcome.

### Limitations

A limitation inherent to all depth electrode studies is limited spatial sampling. Since depth electrodes are only implanted in certain brain areas, the information used to build our networks does not consider possible interictal spikes in non‐sampled regions. This may explain the two (of 22) patients who did not achieve seizure freedom despite having a source spike concordance score > 70%. It is possible that for these patients, there are additional source nodes not sampled by the depth electrodes. Lastly, it is not always possible to delineate an epileptic network for a given patient. For six patients we did not find statistically significant propagation between any two channels and therefore were unable to describe an epileptic network. These patients had to be excluded from our study. Given that SEEG is typically recorded over several days, it may be possible to use longer segments of interictal activity to detect enough interictal spikes from which we can describe a network. Our experience indicates that longer EEG sections are more likely to yield significant networks.

We were unable to demonstrate statistical superiority of any one concordance measure using the McNemar test. This test relies on a large sample size to capture differences between predictive models; it is possible that we were unable to demonstrate superiority of the source spike concordance measure due to the sample size (*n* = 39). If we doubled our sample size while keeping the proportion of true/false positives/negatives the same, the McNemar test would find that source spike concordance is significantly superior to general spike concordance.

While we use two‐fold cross‐validation with 10,000 iterations to account for possible overfitting to the data, increase the generalizability of our measures, and include additionally an independent test set, validation data from another epilepsy center would provide a more definitive answer as to the predictive ability of our measures.

## Conclusion

Epileptic networks based on interictal spike propagation in SEEG may predict seizure freedom in drug‐resistant epilepsy populations. We propose a simple quantitative method to delineate the source of interictal activity, and we demonstrate a strong ability to predict surgical outcome. We find that source spike concordance is a strong predictor of seizure freedom demonstrated by high sensitivity (0.91) and specificity (0.94), and this measure provides a specific approach for the localization of the EZ. Patient‐specific IED propagation networks may supplement other forms of neurological testing during the pre‐surgical evaluation.

## Author Contributions

Abdullah Azeem designed and conceptualized the study, analyzed the data, and drafted the manuscript for intellectual content. Nicolas von Ellenrieder interpreted the data, designed the statistics procedure, and revised the manuscript for intellectual content. Jeffrey Hall, Francois Dubeau, and Birgit Frauscher revised the manuscript for intellectual content. Jean Gotman supervised the project, interpreted the data, and revised the manuscript for intellectual content.

## Conflict of interest

AA, NVE, FD, JH, and JG have no conflicts of interest to disclose. BF reports personal fees from Eisai and UCB and non‐financial support from Eisai and UCB.

## Data Availability

Data may be available in anonymized format by request from the corresponding author.
